# Biomechanical evaluation of tibial bone adaptation after revision total knee arthroplasty: A comparison of different implant systems

**DOI:** 10.1371/journal.pone.0184361

**Published:** 2017-09-08

**Authors:** María Paz Quilez, Belen Seral, María Angeles Pérez

**Affiliations:** 1 M2BE-Multiscale in Mechanical and Biological Engineering, Departamento de Ingeniería Mecánica, Instituto de Investigación en Ingeniería de Aragón (I3A), Universidad de Zaragoza, Zaragoza, Spain; 2 University Clinic Hospital “Lozano Blesa”, Aragón Institute of Health Science (IACS), University of Zaragoza, Zaragoza, Spain; University of Illinois at Urbana-Champaign, UNITED STATES

## Abstract

The best methods to manage tibial bone defects following total knee arthroplasty remain under debate. Different fixation systems exist to help surgeons reconstruct knee osseous bone loss (such as tantalum cones, cement, modular metal augments, autografts, allografts and porous metaphyseal sleeves) However, the effects of the various solutions on the long-term outcome remain unknown. In the present work, a bone remodeling mathematical model was used to predict bone remodeling after total knee arthroplasty (TKA) revision. Five different types of prostheses were analyzed: one with a straight stem; two with offset stems, with and without supplements; and two with sleeves, with and without stems. Alterations in tibia bone density distribution and implant Von Mises stresses were quantified.

In all cases, the bone density decreased in the proximal epiphysis and medullary channels, and an increase in bone density was predicted in the diaphysis and around stem tips. The highest bone resorption was predicted for the offset prosthesis without the supplement, and the highest bone formation was computed for the straight stem. The highest Von Mises stress was obtained for the straight tibial stem, and the lowest was observed for the stemless metaphyseal sleeves prosthesis.

The computational model predicted different behaviors among the five systems. We were able to demonstrate the importance of choosing an adequate revision system and that in silico models may help surgeons choose patient-specific treatments.

## 1. Introduction

The aim of revision knee arthroplasty is to obtain a stable articulation with an acceptable level of pain-free range of motion, by preserving remaining viable bone structures, reconstructing existing bone defects and restoring the joint level [1, 2]. The osseous defects observed in revision total knee arthroplasty (TKA) are challenging to manage and can be underestimated preoperatively. Because the number of the total knee arthroplasties increases in younger and more active patients, the need for revision will continue to increase. There are several classification systems for bone defects. The most commonly used system is the Anderson Orthopaedic Research Institute (AORI) [3]. For the tibia, there are different fixation techniques for bone defects of type 2 (defects in the metaphyseal tibial plateau bone) and type 3 (deficient metaphyseal plateau bone) [3]. These fixation techniques include diaphyseal stem fixation, metal augments, tantalum cones, custom-made implants, allograft reconstruction, and offset tibial stems. [4–8]. However, there is no optimal method for the management of these types of bone defect. Autografts and allografts have been successful with small bone defects [3], but in cases of large bone defects, high failure rates were observed with this solution [9]. Augments or supplements attached to the tibial tray could be a good solution, although wear debris and corrosion has been observed in the short term [10]. Offset stems can solve three problems found in the revision of TKA: gap balancing, anatomical mismatch and malalignment [11]. Finally, metaphyseal sleeves and tantalum cones are good alternatives for large bone defects [10, 12]. Most of these procedures have shown promising early outcomes [6, 7, 12–14] and mid-term results [15, 16] but the long-term effect of bone resorption of the tibia remains unknown.

Several computational studies based on the finite element (FE) method have focused on the bone remodeling effects of femoral [17–20] and tibial prostheses [21–23], but these studies addressed only primary surgeries. Completo et al. [24] developed an FE analysis and an experimental (strain gauge) model of intact and implanted synthetic tibias and experimentally validated their computational approach. Chong et al. [22] analyzed the cementing technique used for tibial fixation and predicted changes in bone apparent density after prosthesis implantation and they concluded that using a hybrid fixation induced the least amount of bone resorption.

Cawley et al. [23] investigated the stress and strain distribution in the proximal tibia for full cementation and surface cementation of a primary tibial component. Their computational and experimental results confirmed that surface cementation resulted in less proximal bone resorption, reducing the possibility of aseptic loosening. Completo et al. [24] experimentally and computationally studied the strain distribution generated by two femoral stems in revision TKA. They concluded that different stem extensions affected the strain behavior of cancellous bone under the tibial tray.

The purpose of this study was to conduct an FE analysis of revision knee tibial implants. The main objectives were to analyze the changes in the tibial bone density and implant Von Mises stresses of five different tibial implant designs and to compare the biomechanics of the metaphyseal sleeves with and without a short stem. Although this topic has been extensively studied, bone remodeling models have not previously been applied to compare different revision knee systems.

## 2. Materials and methods

### 2.1. FE models

**Fig 1:**
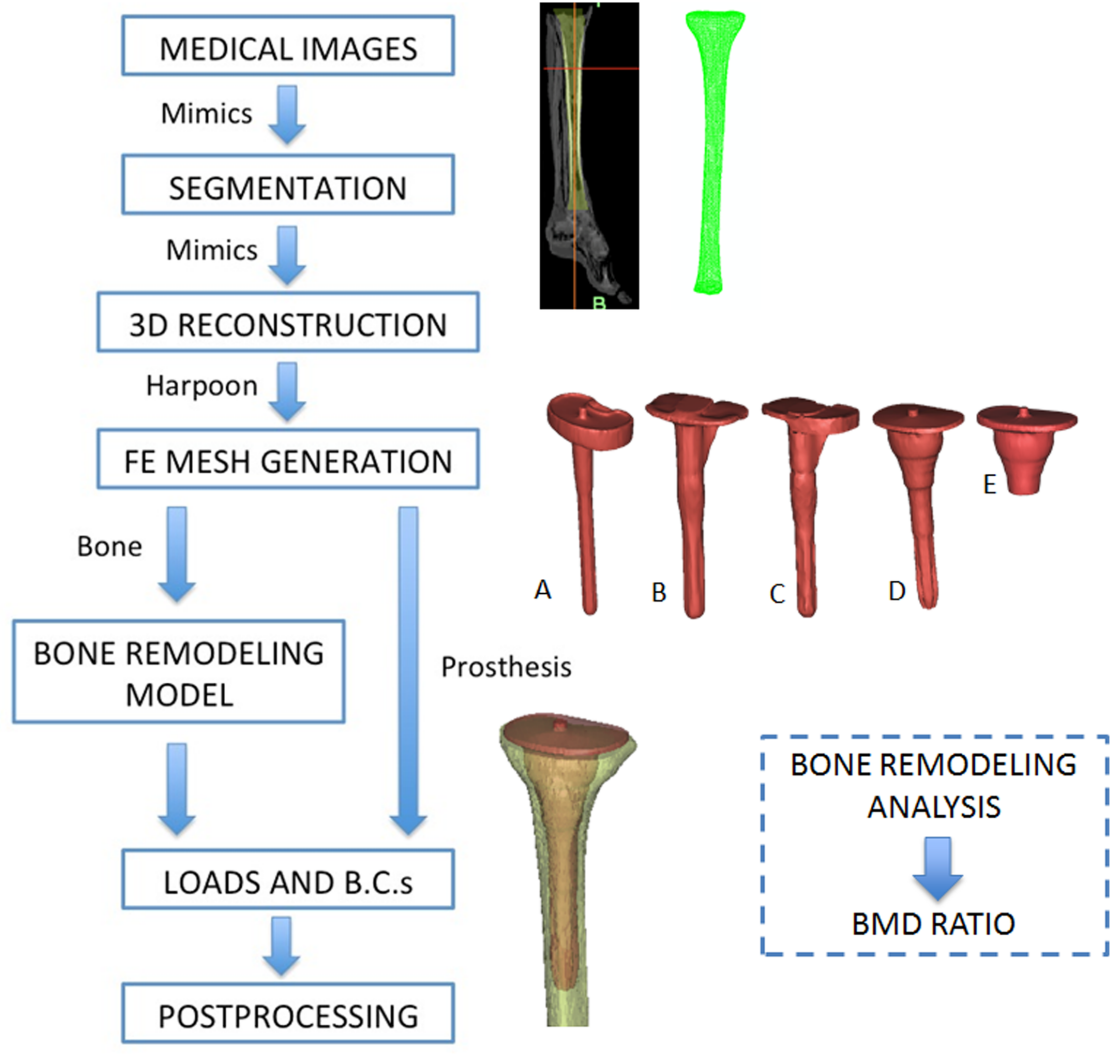
Process followed for the reconstruction and final FE analysis starting with the medical images. (A) Straight stem, (B) offset stem without supplement, (C) offset stem with supplement, (D) sleeves with stem and (E) stemless sleeves.

The scheme showing all the steps followed for the complete reconstruction of the prosthesis modelling until the final FE bone remodeling analysis is presented in Fig 1. First, a human male (56 years old) left tibia and different prostheses were scanned, and the images were stored in Dicom format. Institutional Review Board approval was obtained for this study. The images were acquired using a 64-detector multidetector computerized tomography (MDCT) system (Brilliance 64, Philips Healthcare, Amsterdam, The Netherlands) using a tube current of 257 mA and a voltage of 120 kV. The spatial resolution was 0.65 x 0.65 mm, with a reconstructed matrix of 768 x 768. The slice thickness was 2 mm. All the images were checked to validate their quality and ensure the absence of artifacts in the area of interest. Five different types of prostheses were analyzed: a prosthesis with a straight stem; two prostheses with sleeves, with and without stems (PFC SIGMA TC3, Depuy, Johnson & Johnson, Warsaw, USA); and two prostheses with offset stems, with and without supplements (NexGen Legacy Constrained Condylar Knee-LCCK, Zimmer, Indiana, USA). The five prostheses were manufactured from Cobalt Chromium Molybdenum Alloy (CoCrMo), the stems were uncemented, and the tibial baseplate were fixed with a superficial cement layer.

Mimics v. 17 (Materialise, Leuven, Belgium) was used to segment and reconstruct the geometrical models. Using 3-matic (Materialise, Leuven, Belgium), prostheses were introduced into the healthy tibia and the FE meshes were generated, as shown in Fig 1. Models were meshed using 4-node tetrahedral elements, which were used to reproduce the complex geometry of the bone with sufficient accuracy. The element size used (2 mm) was within the asymptotic region of convergence and represents a good tradeoff between numerical accuracy and computational cost. A linear elastic analysis was performed.

Using Mimics and a calibration phantom, we were able to compute the bone apparent density distribution through the Hounsfield units (HU) obtained from the CT scan. This information was used to validate the results from the bone remodeling simulations. CoCrMo material properties were assigned for the prostheses. A Young’s modulus of 200 GPa was assigned, and the Poisson’s ratio was established at 0.32 [25]. The bone-prosthesis interface was assumed and simulated as completely bonded, and the cement used for proximal tibial plate fixation was neglected because a negligible thickness is used clinically.

### 2.2. Bone remodelling model

A previously developed bone remodelling model was used [26]. Briefly, a damage-based remodeling model was used in which damage was understood as a measure of bone porosity. A no-damage situation corresponds to an ideal situation of null porosity and isotropic conditions, but a damage state is related to bone resorption and an increase in void ratio. Bone formation leads to a decrease in porosity (damage reduction). Additionally, directional mass distribution was considered (Cowin fabric tensor) [27, 28], which considered the porosity and directionality of the trabeculae. Therefore, anisotropic and non-homogeneous bone apparent density distribution was computed. More details on the bone remodeling mathematical model can be found in Pérez et al [29] and Garijo et al [26]. The Young’s modulus and the Poisson’s ratio were related to the bone apparent density, ρ [30]:

$$E=\{\begin{array}{c} 2014\rho^{2.5}\;if\;\rho \leq 1.2g/cm^{3}\\ 1763\rho^{3.2}\;if\;\rho >1.2g/cm^{3} \end{array}$$ (1)

$$\nu =\{\begin{array}{c} 0.2\;if\;\rho \leq 1.2g/cm^{3}\\ 0.32\;if\;\rho >1.2g/cm^{3} \end{array}$$ (2)

For cortical bone, ρ = 1.92g/cm^3^, and the Young’s modulus equals 10,287 MPa.

### 2.3. Loads and boundary conditions

**Fig 2:**
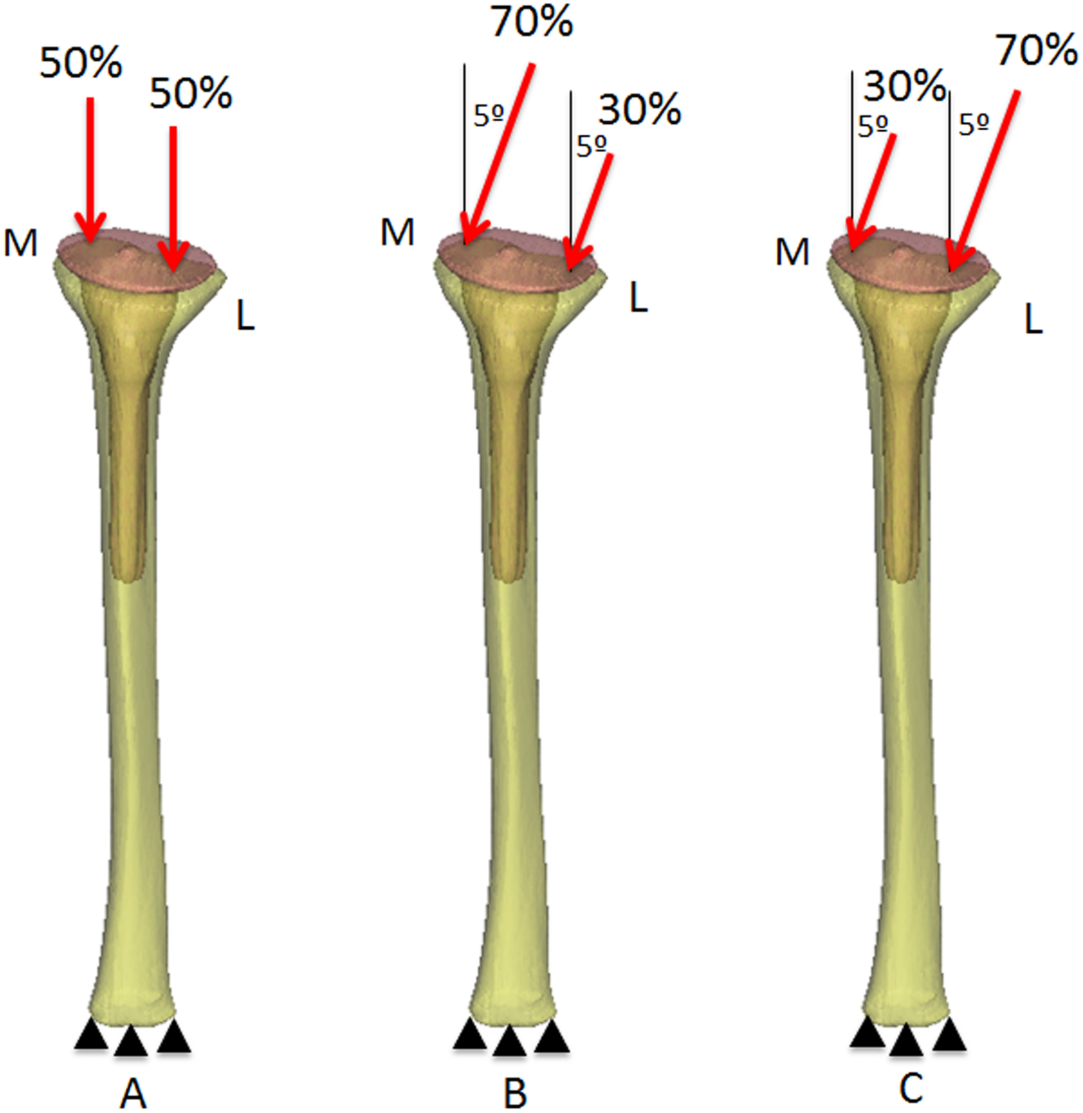
Boundary and loading conditions (M: medial and L: lateral).

Loading conditions for the tibia were previously used by Pérez et al. [29]. Therefore, a summary of them is presented below. Distally, the tibial diaphysis was fixed in the vertical and horizontal directions. Physiological-like loading conditions were simulated. Loading conditions were simulated through the joint reaction force at the condylar surface [31, 32]. Specifically, walking movement was considered to be represented by three main load cases, which were iteratively repeated (Fig 2). The first load case corresponded to the joint reaction force equally distributed in the two tibial condyles (vertical direction) (Fig 2A). In the second load case, the joint reaction force was distributed across the medial and lateral condyles at 70% and 30%, respectively. Finally, in the third load case, the joint reaction force was distributed across the medial and lateral condyles at 30% and 70%, respectively. In the second and third load case, the force was inclined 5° from the vertical direction, so a horizontal force appeared medially (Fig 2B and 2C). The load values corresponded to a body weight of 70 kg. Loads were applied through a rigid surface covering the lateral and medial condyles. Therefore, the loads were uniformly distributed over the condyles. The load values considered for the tibia are represented in Table 1.

**Table 1:** Values of applied forces (N) at the tibia (with permission of Pérez et al., 2010).

		Medial condyle		Lateral condyle	
Case	Cycles/day	X-axis	Z-axis	X-axis	Z-axis
1	3000	0.0	-1062.08	0.0	-1062.08
2	500	-129.6	-1353.28	-55.68	-634.88
3	500	55.68	-634.88	129.6	-1353.28

### 2.4. Numerical simulation

The FE analyses were performed using Abaqus v6.13 (Dassault Systemes Simulia Corp., Providence, RI, USA, 2006) for the tibia (Fig 1), with the bone material properties assigned using a user routine containing a previously described numerical model described previously (section 2.2). Most of the bone remodeling simulations started from an initial situation where the whole bone apparent density distribution was 0.5g/cm^3^ and was isotropically distributed. Then, the loading conditions (section 2.3) were iteratively applied, changing the bone apparent density distribution (value and directionality in every integration point). Finally, a non-homogeneous and anisotropic bone apparent density distribution was predicted. The non-uniform final density distribution was generally considered to have been achieved (i.e., the bone remodeling analysis was finished) when the total change in the bone apparent density in the whole tibia, *e*, was lower than a threshold limit, *e*_*lim*_.

$$e=\frac{\int \left(\Delta \rho \right)dV}{\int dV}\leq e_{\text{lim}}$$ (3)

where Δρ is the change in the bone apparent density and *V* is the tibia volume. We set *e*_*lim*_ = 2x10^-4^ [33]. After convergence, we compared the bone apparent density distribution predicted with that computed based on the CT data. For this comparison, the following relationship was used to compute the bone apparent density from the CT data (HU) (ρ = 1+7.185 x 10^−4^ HU). This relationship originated from the calibration phantom used. This comparison will validate the bone remodelling model used.

## 3. Results

### 3.1. Validation of the bone remodeling model

**Fig 3:**
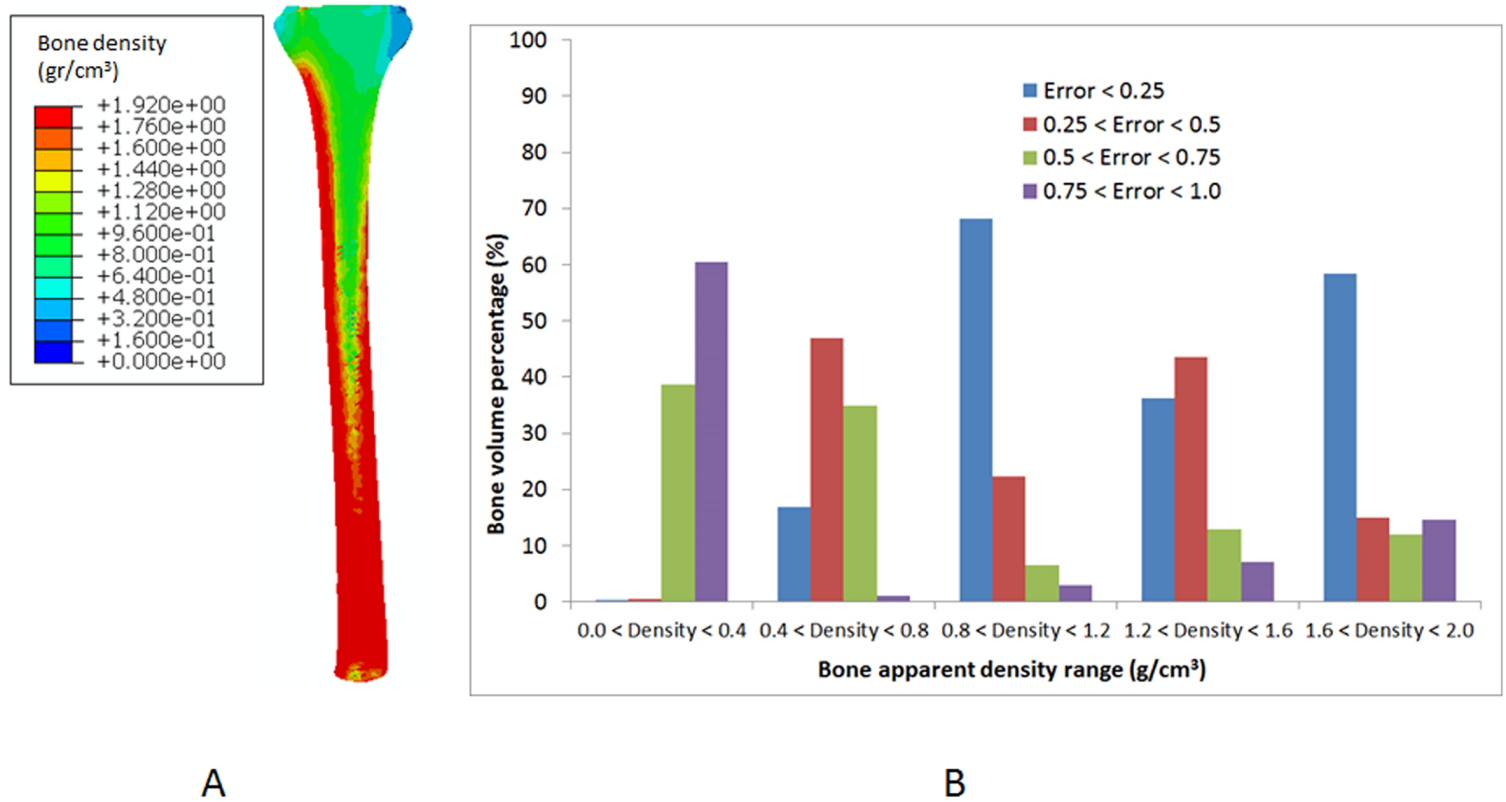
Results obtained from the comparison of HU based on CT data and bone apparent density distribution predicted using the bone remodeling model. (A) Bone apparent density distribution predicted using the bone remodeling model. (B) Percentage of bone volume with a certain error level for four different density ranges in the tibia (with permission of Pérez et al., 2010).

Bone remodeling predictions before prosthesis implantation are shown in Fig 3A. Using the calibration phantom relationship (section 2.4), the bone apparent density was obtained from the HU values for the tibia. The predicted bone apparent density and the value obtained from the HU were compared and the relative error between them was computed and presented in Fig 3B. Pérez et al. [29] performed a similar validation. For each bone apparent density range, the relative error between the predicted bone apparent density and the value computed from the HU was calculated, and then the percentage of bone volume with this specific relative error was presented (Fig 3B). The cortical regions were accurately predicted, and most of the cortical bone volume was under a relative error lower than 25%. In contrast, the most important differences were estimated for the trabecular bone (Fig 3B).

The results allowed us to validate the bone density predictions and, thus, compute the long-term bone behavior after prosthesis implantation.

### 3.2. Long-term bone density predictions after prosthesis implantation

**Fig 4:**
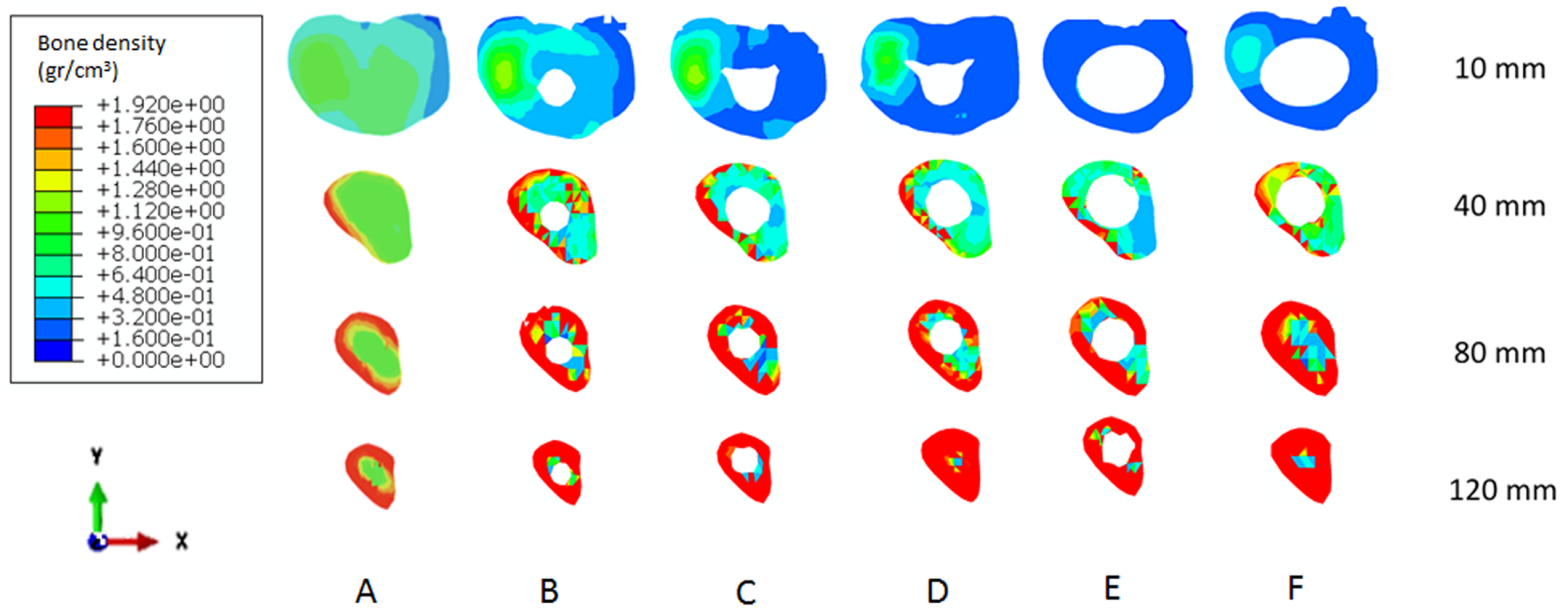
Axial cross-sectional views of the bone density distribution (gr/cm^3^) at 10, 40, 80 and 120 mm from the top of the tibial tray, 400 days after analysis. (A) Before implantation. After prosthesis implantation: (B) straight stem, (C) offset stem with supplement, (D) offset stem without supplement, (E) sleeves with stem, and (F) stemless sleeves. (See the various prosthesis models in Fig 1).

The bone density distributions predicted after prosthesis insertion are presented in Fig 4 (after 400 days). In all cases, the bone density decreases in the proximal epiphysis, and an increase in the bone density is predicted in the diaphysis and at the bone around the stem tips. Qualitatively, the sleeves with stem prosthesis generated high bone resorption in the proximal epiphysis, followed by the stemless methaphyseal sleeves prosthesis (Fig 4E and 4F). The prosthesis with both sleeves and a stem produced the highest value of bone formation around the stem.

**Fig 5:**
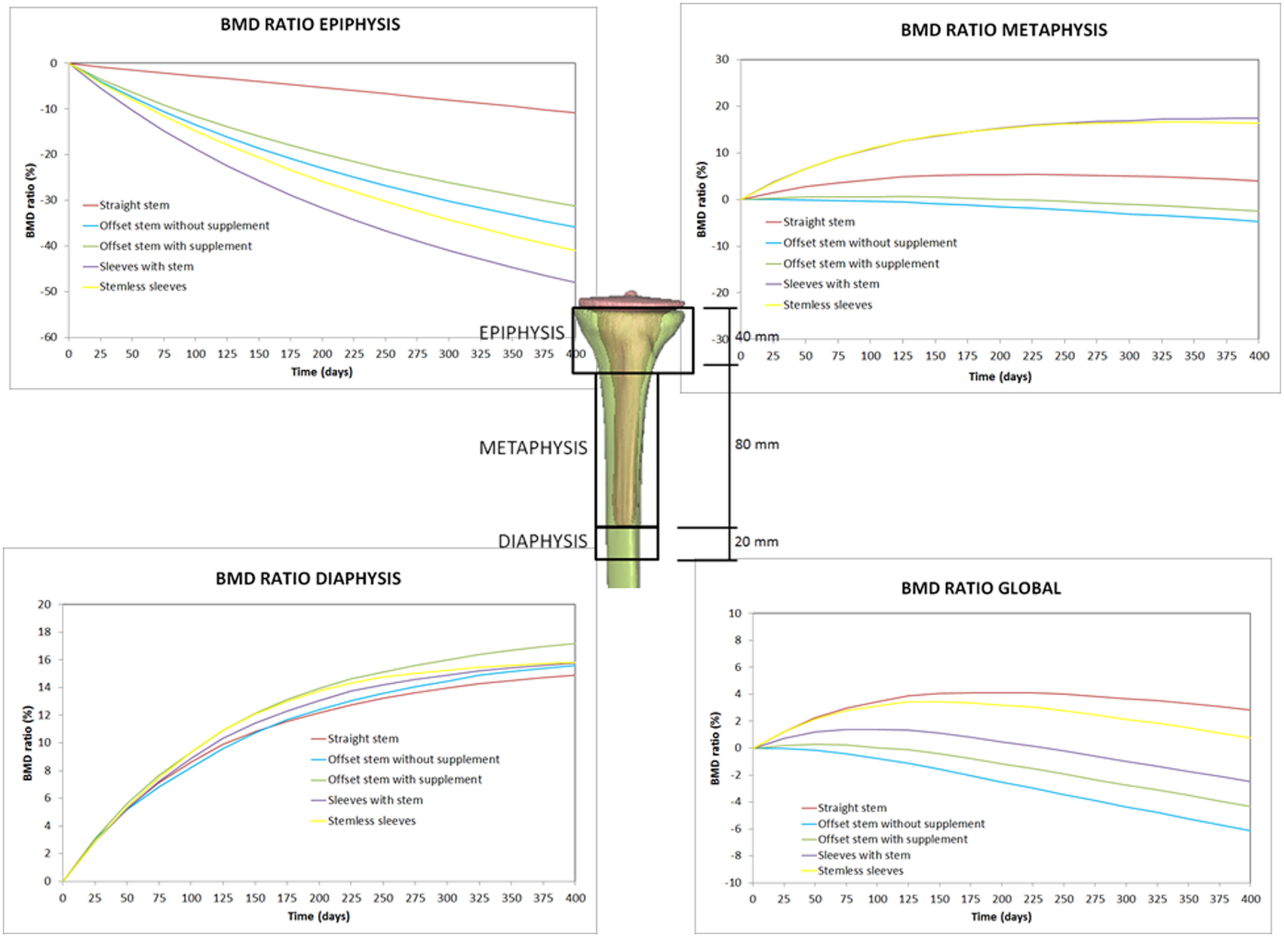
Bone mineral density ratio variation in different regions of interest (ROIs).

The predicted bone density distribution presented in Fig 4 was quantified in Fig 5, and the bone density ratio (BMD) was computed for the five prostheses. The BMD ratio was calculated at every time increment as the difference between the actual bone density distribution and the bone density distribution just after prosthesis implantation (time = 0). Three regions of interest (ROIs) were selected for measurements of the BMD of the tibia [34] (Fig 5): the epiphysis, the metaphysis and the diaphysis (the area around the stem tip). High bone resorption was predicted at the epiphysis for the five prostheses (Fig 5). The highest bone resorption was computed for the sleeves with stem prosthesis, followed by the stemless sleeves prosthesis. The lowest bone resorption was computed for the straight stem, followed by the two offset stem prostheses. At the metaphysis region, all five prostheses generated bone resorption, although in the short term, small formation was predicted for the straight and stemless sleeves prostheses. In the long term, the highest bone resorption was obtained for both offset stems. The lowest bone resorption was computed for the straight stem, followed by the stemless sleeves and the sleeves with stem prostheses. At the diaphysis region, bone formation was predicted for all prostheses. An important difference was observed between the two prostheses with sleeves compared with the other tested prostheses. After 400 days, the highest bone formation was predicted for the stemless sleeves and sleeves with stem prostheses, whereas the lowest bone formation was computed for the straight stem, offset stem with supplement and offset stem without supplement prostheses. Globally, the highest bone resorption (-6.1%) was predicted for the offset prosthesis without supplement, followed by the offset prosthesis with supplement (-4.3%); 2.4% bone resorption was predicted for the sleeves with stem prosthesis. Bone formation was globally predicted for the stemless sleeves (0.7%) and for the straight stem (2.8%) (Fig 5).

**Fig 6:**
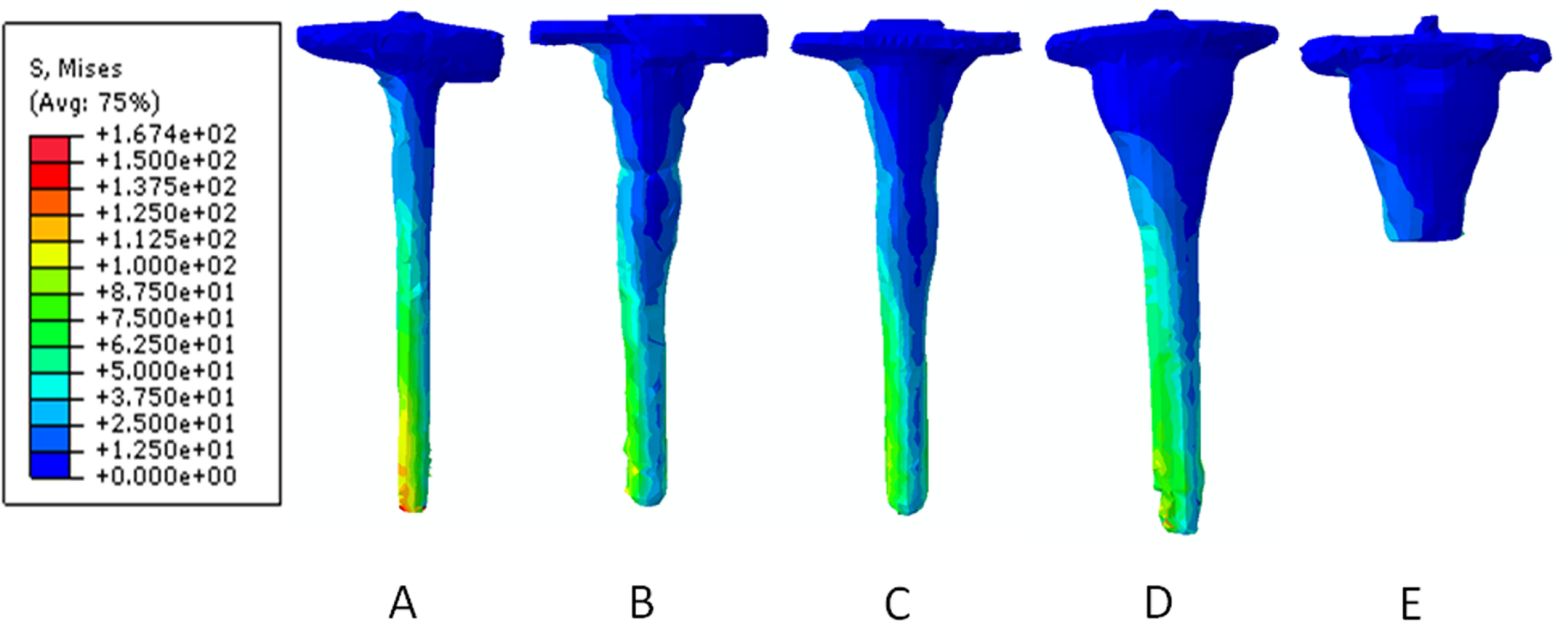
Von Mises stress distribution for each prosthesis at the end of the simulation: (A) straight stem, (B) offset stem with supplement, (C) offset stem without supplement, (D) sleeves with stem, and (E) stemless sleeves.

Maximum values of implant Von Mises stress are shown in Table 2. None of the prostheses reached the material yield strength (450 MPa). The distribution of Von Mises stress is presented in Fig 6. The highest value was obtained for the straight stem prosthesis, and the lowest was obtained for the stemless sleeves prosthesis. The peak Von Mises stress was located along the stem, mainly at the stem tip.

**Table 2:** Maximum values of Von Mises stress of the prostheses.

Prosthesis	Straight stem	Offset stem with supplement	Offset stem without supplement	Sleeves with stem	Stemless sleeves
VM Stress (MPa)	166.3	119.1	104.0	125.7	49.22

## 4. Discussion

Innovations in the implant systems designed for revision TKA have provided surgeons with many tools to address the complex challenges associated with revision surgery. Implant selection should be based on the severity of bone loss and the status of the ligamentous and soft tissue stabilizing structures [14]. Biomechanical studies based on the FE method, such as the one developed here, may be an innovative tool to predict the long-term behaviors of TKA revision systems as it has been used for total hip arthroplasty (Exeter and metal-on-metal hip resurfacing prostheses) [19, 20]. We also need to further understand the biomechanics of revision TKA. In vitro tibial models [35], experimental studies and FE studies [21, 24, 36–38] may help us achieve this goal. Additionally, studies such as the one presented here can be complementary to short-term or mid-term clinical results [3, 6–8, 10–12, 14–16, 39–41]. Different reconstruction techniques of bone defects can be used in TKA revisions [3, 10–12, 41]. Vasso et al. reviewed previous studies of revision TKA suing different solutions and the mean clinical follow-up was 4.7 years [41]. Barnett et al followed up 51 who had received stepped porous titanium metaphyseal sleeves for 4 years. Therefore, long-term results are needed in addition to the short- and mid-term results in order to form conclusions.

Long stems seemed to work very well in the long-term (Fig 5). The straight stem globally predicted a positive BMD ratio (bone formation) and in the diaphysis. Haas et al reported excellent mid-term results in 84% of patients using long stems [42]. However, bone loss increased proximally in most of the reconstruction techniques (Fig 5). Our computational study estimated bone resorption at the epiphysis in the five systems analyzed (Figs 4 and 5). For the sleeves with stem and stemless metaphyseal sleeves prostheses, the highest bone resorption at the epiphysis was predicted because although sleeves improve the rotational stability, they off-load the epiphysis [41].

Among other advantages, the use of an offset stem seems to facilitate implant alignment. However, no long-term clinical results in terms of bone resorption and bone formation have been reported in the literature [11]. From our results, long-term predictions are not quite satisfactory. Our calculations globally estimated bone resorption (Fig 5) and the other systems analyzed generated better results in the regions analyzed. This result could be due to changes in stem alignment, which critically modify the stress distribution within the tibia.

A number of limitations of our research warrant discussion. One limitation is related to the load values. We assumed mean values for joint contact forces. The application points were also an approximation of reality because the tibia considered here was different from that used in the loading reference studies [29, 31, 32]. Different load sequences were tested, and the same results as those reported in the Results section were obtained. Therefore, no relationship existed between the sequences in which the loads were applied. However, newly published research has shown that patient-specific loads can be predicted from the bone density distribution which could improve this limitation [43]. The use of an Artificial Neural Network (ANN)-based approach is promising not only for loading prediction but also formultiscale bone remodeling simulation [44–46]. Another limitation related to the assumed loads is that we analysed only walking loads and neglected other activities [47–50]. A further limitation is that a perfectly osseointegrated bone–implant interface was assumed. Thus, we neglected the initial situation immediately after prosthesis implantation. In the future, a bone-implant osseointegration model could be developed to model the adhesion between the implant and the bone [25, 51] allowing bone ingrowth and damage to be simulated simultaneously. A single tibia model was used to perform this analysis. In the authors’ opinions, however, this limitation does not reduce the importance and generality of the obtained results. These types of simulations have high computational costs, but new methodologies to accelerate bone remodeling predictions may be applied to reduce the computation time [33]. Finally, initial tibial bone defects were not simulated before prosthesis implantation. The incorporation of the bone defects will slightly modify the results obtained in the short term, but in the long term, the results would have been very similar. Bone adaptation would have been slightly different at the first time increments, but as the simulation evolves, bone adapts depending on the loading conditions. Thus, in the long term, almost no differences would have been predicted.

All the limitations described above must be considered when attempting to draw conclusions from this study. Bony defects around tibial implants are common during revisions. Metaphyseal filling sleeves are an alternative to allografts. The solid metaphyseal fixation of the sleeves leads to the highest proximal bone resorption. However, bone formation was globally predicted for the stemless metaphyseal sleeves and the straight stem, with BMD ratios of 0.7% and 2.8%, respectively.

All the technical difficulties associated with the use of tibial stems in revision TKA could be avoided if adequate stability of the construct could be obtained without using stems. The highest value of Von Mises stress was obtained for the straight stem prosthesis, and the lowest was obtained for the stemless sleeves prosthesis (Fig 6). The peak Von Mises stress was located along the stem, mainly at the stem tip. Long-term data are needed to determine where these new implants fit within the currently available methods. Bone remodeling models are useful tools for the biomechanical comparison of implants and allowed us to predict their long-term behaviors. Although bone remodeling simulations are not novel, their application in this study to revision knee systems allowed us to quantitatively and qualitatively compare multiple systems. Based on this bone remodeling model, we can conclude that revision TKA systems produce bone resorption in the epiphysis and metaphysis regions, although bone formation is predicted in the diaphysis, and important differences exist among the different systems. Therefore, this predictive tool may aid in the surgeon’s treatment decision and in the development of patient-specific treatments.
